# *Brucella spp *noncanonical LPS: structure, biosynthesis, and interaction with host immune system

**DOI:** 10.1186/1475-2859-5-13

**Published:** 2006-03-23

**Authors:** Patrícia Gomes Cardoso, Gilson Costa Macedo, Vasco Azevedo, Sergio Costa Oliveira

**Affiliations:** 1 Department of Biochemistry and Immunology, Biological Sciences Institute, Federal University of Minas Gerais, Belo Horizonte-Minas Gerais, Brazil; 2 Department of General Biology, Biological Sciences Institute, Federal University of Minas Gerais, Belo Horizonte-Minas Gerais, 30161-970, Brazil

## Abstract

*Brucella spp*. are facultative intracellular pathogens that have the ability to survive and multiply in professional and non-professional phagocytes, and cause abortion in domestic animals and undulant fever in humans. Several species are recognized within the genus *Brucella *and this classification is mainly based on the difference in pathogenicity and in host preference. *Brucella *strains may occur as either smooth or rough, expressing smooth LPS (S-LPS) or rough LPS (R-LPS) as major surface antigen. This bacterium possesses an unconventional non-endotoxic lipopolysaccharide that confers resistance to anti-microbial attacks and modulates the host immune response. The strains that are pathogenic for humans (*B. abortus*, *B. suis*, *B. melitensis*) carry a smooth LPS involved in the virulence of these bacteria. The LPS O-chain protects the bacteria from cellular cationic peptides, oxygen metabolites and complement-mediated lysis and it is a key molecule for *Brucella *survival and replication in the host. Here, we review i) *Brucella *LPS structure; ii) *Brucella *genome, iii) genes involved in LPS biosynthesis; iv) the interaction between LPS and innate immunity.

## Background

*Brucellae *are Gram-negative cocccobacilli, facultative intracellular bacterial pathogens of both humans and animals. The bacteria penetrate the mucosa of the nasal, oral, or pharyngeal cavities and are phagocytized by host macrophages, where survival and replication occurs. Brucellosis is a zoonotic disease that is difficult to diagnose and treat that causes heavy economic losses and human suffering, characterized by undulant fever that, if untreated, can develop into a chronic infection with symptoms persisting for several months. Chronic infections may result in infection of secondary tissues, including heart and brain. Symptoms may also recur years after the original infection. The pathological manifestations of brucellosis are diverse and include arthritis, endocarditis, and meningitis in humans, while animal brucellosis is characterized by spontaneous abortion [1]. Six species are recognized within the genus *Brucella*: *B. abortus*, *B. melitensis*, *B. suis*, *B. ovis*, *B. canis*, and *B. neotomae*. This classification is mainly based on the difference in pathogenicity and in host preference [2]. The main pathogenic species worldwide are *B. abortus*, responsible for bovine brucellosis; *B. melitensis*, the main etiologic agent of ovine and caprine brucellosis, a disease that causes abortion in ewes and goats resulting in huge economic losses, particularly in Mediterranean countries and *B. suis *responsible for swine brucellosis. *B. abortus *infection is acquired by humans through contact with infected livestock and consumption of unpasteurized dairy products. *B. ovis *and *B. canis *are responsible for ram epididymitis and canine brucellosis, respectively [3]. For *B. neotomae *only strains isolated from desert rats have been reported. *Brucella *strains have also been isolated from a great variety of wildlife species such as bison, elk, feral swine, foxes, hares, African Buffalo, reindeer, and caribou [4]. Recently, two new species have been proposed to be added to this genus, *Brucella cetaceae *and *Brucella pinnipediae *isolated from marine mammals, cetaceans and pinnipeds, respectively [5,6]. Distinction between species and biovars is currently performed by differential tests based on phenotypic characterization of lipopolysaccharide antigens, phage typing, dye sensitivity, CO_2 _requirement, H_2_S production, and metabolic properties [7].

Additionally, *Brucella *can be used as a biological weapon since transmission through a spray is possible, as has been reported with human contamination during abortion of infected animals or bacterial spraying in laboratories [8]. The bacteria is highly contagious and it is suggested that 10 to 100 bacteria would be sufficient to produce a contaminating spray for humans. Several countries have been suspected of studying the agent as a biological weapon, but to date, no use of *Brucella *in a bioterrorist attack has been reported.

In this article, we review the importance of the lipopolysaccharide in *Brucella *virulence, discussing the LPS chemical composition, the *Brucella *genome, the genes involved in LPS biosynthesis, and the interaction between LPS and innate immunity.

## Chemical composition of lipopolysaccharide from different *Brucella *strains

**Figure 1:**
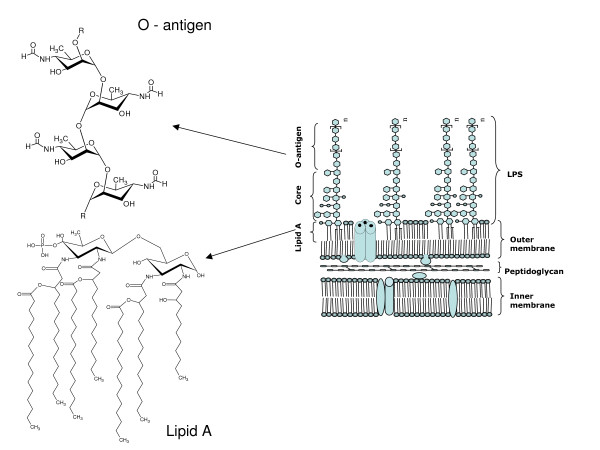
Schematic structure of lipopolysaccharide (LPS) from *Brucella spp.*

Several studies on virulence factors were directed at the main components of the outer membrane. The outer membrane contains the lipopolysacharide (LPS) that is the *Brucella *major virulence factor. Lipopolysaccharide is vital to both the structural and functional integrity of the Gram-negative bacterial outer membrane. Ubiquitously expressed by all Gram-negative bacteria, and containing several well-conserved domains, LPS also serves as one of the primary targets of the innate arm of the mammalian immune system. *Brucella *possesses a peculiar non-classical LPS as compared with classical LPS from enterobacteria such as *Escherichia coli *[9]. The LPS was identified as a major virulence determinant of *Brucella *recognized for its role in virulence when naturally occurring isolates lacking LPS showed reduced survival. LPS has three domains: lipid A, the core oligosaccharide, and the O-antigen or O-side chain (Figure 1). The O-polysaccharide of smooth-type *Brucella *LPS (S-LPS) is an unbranched homopolymer of 1,2-linked4,6-dideoxy-4-formamido-α-D-mannopyranosyl usually with an average chain length of 96 to 100 glycosyl subunits [10]. The O-polysaccharide is linked to a core oligosaccharide composed of mannose, glucose, 2-amino-2,6-dideoxy-D-glucose (quinovosamine), 2-amino-2-deoxy-D-glucose (glucosamine), 3-deoxy-D-manno-2-octulosonic acid (KDO) and unidentified sugars. The lipid A, linked to the core oligosaccharide, contains 2,3-diamino-2,3-dideoxy-D-glucose (diaminoglucose) as backbone, amide and ester-linked long chain saturated (C_16:0 _to C_18:0_) and hydroxylated (3-OH-C_12:0 _to 29-OH-C_30:0_) fatty acids [11]. The hydrophobic lipid A region constitutes mostly the outer coating of the outer membrane and is responsible for many of the endotoxic properties attributed to LPS [12]. Thermotropic phase behaviour [13] and immunochemical analysis [14] of *B. abortus *and *B. melitensis *lipid A suggest a disaccharide backbone molecule linked in a β1–6 configuration. Ethanolamine, neutral sugars and ester-linked acyl-oxyacyl fatty acids are not found, and phosphate is absent or present in reduced quantities [11]. *Brucella *lipid A contains strongly bound outer membrane protein fragments that are not removed by conventional procedures used to release the lipid-A-associated protein of enterobacterial LPS [11,14]. The heterogeneity of enterobacterial LPS is well known to be related to the length of its O-polysaccharide and different chemical substitutions in the core oligosaccharide and lipid A [15]. In enterobacterial lipid A, the degree of heterogeneity depends upon the different combinations in which amide and ester-linked fatty acids, phosphate, neutral sugars, ethanolamine and different types of backbone amino sugars occur in the molecule [16]. In *Brucellae *lipid A, the degree of heterogeneity may depend mainly on the various fatty acid substitutions. The absence of backbone constituents (other than lipids) and ester-linked acyl-oxyacyl residues in *Brucella *lipid A might account for the restricted number of variants as compared to enterobacterial lipid A. Determination of the intrinsic heterogeneity in *Brucella *LPS is important to explain in a more realistic perspective its chemical nature and biological behavior. For practical purposes, this is significant, since LPS is the most relevant antigen during infection and vaccination. In addition, LPS and LPS-related molecules are extensively used in immunological studies and in the diagnosis of brucellosis [17].

## *Brucella *genomes

Recently, the genome sequences of the *B. melitensis*, *B. suis *and *B. abortus *became available [18-20]. The genomes of *B. suis*, *B. melitensis*, and *B. abortus *are very similar in sequence, organization, and structure. Few fragments are unique among the genomes [20]. Although many aspects of its biology remain to be understood, the sequencing and annotation of its genome paved the way for a highly comprehensive and rapid analysis of its proteome. Comparative genomics provide insights into aspects of *Brucella *virulence that were only suspected before. The era of post-genomic technology offers new and exciting opportunities to understand the complete biology of different *Brucella *species. At the proteome level, extensive metabolic differences were found between the *B. melitensis *reference strain 16 M and its vaccine strain Rev 1. Similarly, laboratory grown *B. melitensis *can be distinguished from *B. abortus *by just looking at their proteome [21]. Using the complete genome sequence of *Brucella melitensis*, Dricot *et al. *[22] generated a database of protein-coding ORFs and constructed an ORFeome library of 3091 Gateway entry clones, each containing a defined ORF. This first version of the *Brucella *ORF (Version 1.1) provides the coding sequences in a user-friendly format amenable to high-throughput functional genomic and proteomic experiments, as the ORFs are conveniently transferable from the entry clones to various expression vectors by recombinational cloning. The cloning of the *Brucella *ORFeome v.1.1 should help to provide a better understanding of the molecular mechanisms of virulence, including the identification of bacterial protein-protein interactions, but also interactions between bacterial effectors and their host targets.

The genome of *B. melitensis *strain 16 M contain 3,294,935 bp distributed over two circular chromosomes of 2,117,144 bp and 1,177,787 bp encoding 3,197 open reading frames (ORFs). The origins of replication of the two chromosomes are similar to those of other proteobacteria. Housekeeping genes, including those involved in DNA replication, transcription, translation, core metabolism, and cell wall biosynthesis, are distributed on both chromosomes [21]. Screens for transpositional mutants attenuated in infection models yielded 184 mutants, suggesting that these genes have a function in the infection process [23]. The *B. suis *1330 genome consists of two circular chromosomes of 2,107,792 bp and 1,207,381 bp [19]. A total of 2,185 and 1,203 ORFs were identified on chromosomes I and II, respectively. Comparison of the *B. suis *genome with that of the *B. melitensis *genome revealed extensive similarity and gene synteny [21]. The majority (>90%) of *B. suis *and *B. melitensis *genes share 98–100% identity at the nucleotide level. The more variable genes (<95% identity) consist primarily of hypothetical genes, as well as a *UreE *urease component, and probable surface-exposed genes such as outer membrane proteins, membrane transporters, a putative invasin, and ShdA-like adhesins. These more variable genes may significantly contribute to the differences in pathogenicity or host preference between these two organisms. The high degree of similarity between the *B. suis *and *B. melitensis *genomes at both the gene and nucleotide level is consistent with the proposition that *Brucella *species should be grouped as biovars of a single species.

The genome of *B. abortus *biovar 1 (Strain 9-941) have 3.3 Mb and is composed of two circular chromosomes of 2,124,242 (Chr I) and 1,162,780 bp (Chr II) [20]. The chromosome sequences of *B. abortus *9-941 were assigned the same strand orientation and origin as those of *B. suis*. The *B. abortus *genome contains 3,296 ORFs annotated as genes, 2,158 on Chr I and 1,138 an Chr II. This is similar to the annotated ORF counts for *B. suis *(3,388) and *B. melitensis *(3,197). The genome of *B. abortus *shared more fragments with *B. suis *and *B. melitensis *than *B. suis *and *B. melitensis *did with each other. *B. abortus *shared more fragments with *B. melitensis *than *B. suis*. Two fragments shared by *B. suis *and *B. melitensis *were not found in *B. abortus*. A 2,774-bp fragment encoding a probable surface protein and two partial ORFs with homology to the insertion sequences IS*711 *and IS*Bm1 *is missing from *B. abortus*. The second fragment is a 25-kb sequence that may be involved in polysaccharide synthesis and was predicted by Vizcaino *et al. *[24] to potentially affect phenotypes of brucellae, such as host preference.

## Genes involved in LPS biosynthesis

The majority of studied genes and their products involved in *Brucella *LPS biosynthesis are those related to O-chain synthesis (Table 1). The large number of attenuated mutants with a structural defect in their lipopolysaccharide (LPS) confirms the importance of this molecule in *Brucella *virulence. In spite of the importance of LPS in the *Brucella *life cycle, very little is known about the metabolic pathways and enzymes required to synthesize it. Certain strains carry mutations in the genes involved in O-chain biosynthesis and attachment and are termed 'rough mutants'(R) strains to differentiate them from the wild-type 'smooth' (S) strains that express O-chain-bearing LPS. Historically, the search for rough mutants was performed in two ways: by a spontaneous phenomenon known as phase variation, in which rough variants appear in culture by a mechanism not well understood [7], or by successive passages in different culture media with searching for spontaneously rough phenotypes. These procedures gave a high number of rough mutants. All of them are avirulent in animal models, but in most cases the parental strain is not available. *B. abortus*, *B. melitensis*, and *B. suis *strains may occur as either smooth or rough, expressing smooth LPS (S-LPS) or rough LPS (R-LPS) as major surface antigen, while *B. ovis *and *B. canis *are two naturally R species, expressing R-LPS as major surface antigen. Generally these mutants are less virulent than the wild type, with the exception of those of *Brucella ovis *and *B. canis*, which are rough but virulent. It is accepted that rough mutants are more sensitive to lysis mediated by complement, and probably this is the main reason why most rough variants have an avirulent phenotype in animal models. To date the question about the capacity of rough mutants to replicate intracellularly is not solved. Some authors have reported that smooth LPS is essential for intracellular survival [25], for example, the vaccine strain RB51 exhibits loss of virulence and cannot replicate within macrophages [26]. On the other hand, there are some reports in which genetically characterized rough mutants did not loose the capacity to replicate intracellularly despite the total absence of the O-antigen [27]*B. abortus *strain RB51 is a stable rough (R) mutant derived from the standard smooth (S) virulent strain 2308. This rough mutant strain that does not contain the O-antigen (O polysaccharide chain of the smooth LPS) is attenuated in its virulence compared to their smooth virulent parental strain [27]. Even though the *Brucella *RB51 rough strain has been widely used as a live vaccine, it induces lower protection compared to the smooth vaccine strain S19 [28]. At similar concentrations, the *Brucella *vaccine strain S19 induces higher protection than the R vaccine strain (RB51). The lack of protective antibodies to the O-side chain of the LPS in animals immunized with *B. abortus *RB51 may explain in part why the R strain induces lower protection against infection [29]. A major problem in animal vaccination against brucellosis with currently used live attenuated smooth *Brucella *strains, such as *B. melitensis *strain Rev.1 for sheep and goats and *B. abortus *strain S19 for cattle, is the fact that vaccinated animals cannot be clearly differentiated from infected animals by the current serological tests. Further, these strains may induce abortions when used in pregnant animals and are virulent for humans. These tests are indeed based on detection of antibody to S-LPS that is immunodominant in the serological responses of both infected and vaccinated animals.

**Table 1:** Genes encoding O-antigen biosynthesis in *Brucella spp.*

Gene	Product
Gmd	GDP-mannose dehydratase
Per	Perosamine synthetase
Pgm	Phosphoglucomutase
Pmm	Phosphomannomutase
ManB	Mannose isomerase
ManC	Mannose guanylyltransferase
Wzm	O-antigen export permease
Wzt	ATP-binding protein
WbkB	no similarity to known genes
WbkC	Methionyl tRNA formyltransferase
WbkA	N-formyl-perosaminyltransferase

Below, we describe the main genes involved in LPS biosynthesis and the role of their encoding products.

### Perosamine synthetase(per)

Godfroid *et al. *[30] described molecular analysis of the genes required for the synthesis of the O-antigen of *Brucella melitensis *16 M. The perosamine synthetase gene was cloned and sequenced. In *V. cholerae *O1, perosamine is synthesized from fructose 6-phosphate via four intermediates: mannose 6-phosphate, mannose 1-phosphate, GDP-mannose, and 4-keto-6-dideoxymannose. Ultimately, this final product is converted to GDP-perosamine by the perosamine synthetase [31]. Because the last step of the perosamine synthesis pathway is identical for *V. cholerae *and *B. melitensis*, it was assumed that the earlier steps might be similar or identical for these two organisms. In *Brucella*, the GDP-perosamine would then serve as a substrate for the addition of a formyl group and could then be polymerized into the O-antigen, translocated to the periplasm, transferred to the lipid A-core oligosaccharide, and exported to the cell surface. The disruption of *per *(B3B2 mutant) totally disabled the O-side chain biosynthesis of *B. melitensis *16 M. The mutation was recreated by gene replacement, indicating that the mutant phenotype was due to the transposon insertion rather than to spontaneous mutation [30]. Indeed, such a disruption prevented any O-side chain production, not only at the surface but also in the cytoplasm of the bacteria indicating that the mutation does not affect the transport of the O-side chain to the outer membrane but does affect an earlier stage of biosynthesis.

### Phosphomannomutase (pmm or manB)

Allen *et al. *[27] to better characterize the role of O-antigen in virulence and survival used transposon mutagenesis to generate *B. abortus *rough mutants defective in O-antigen presentation. A mutant strain was characterized by a truncated rough LPS and DNA sequence analysis of this mutant revealed a transposon interruption in the gene encoding phosphomannomutase (*pmm *or *manB*), suggesting that this activity may be required for the synthesis of a full-length core polysaccharide in addition to O-antigen. This gene is responsible for the interconversion of mannose-6-phosphate and mannose-1-phosphate. In *Brucella*, mannose is both an important precursor in the O-antigen biosynthetic pathway and in the production of the inner core moiety of LPS [32].

### Mannosyltransferases (wbkA, WbdA, B and C)

*B. abortus *genes involved in chronic infection were identified by assessing the ability of 178 signature-tagged mutants to establish and maintain persistent infection in mice [33]. Each mutant was screened for its ability to colonize the spleens of mice at 2 and 8 weeks after inoculation. A mutant with defects in establishing chronic infection which carried a transposon insertion in a *B. abortus *homologue of *Brucella melitensis wbkA*, encoding a N-formyl-perosaminyltransferase that functions in the biosynthesis of O-antigen, was the most highly attenuated. In *E. coli *O9a polysaccharide is polymerised by the action of three different mannosyltransferases WbdA, B and C [34]. In this scheme, WbdC transfers a mannose to the endogenous acceptor (GlcNAc-pyrophosphoundecaprenol). This reaction initiates the growth of the polysaccharide chain and provides the acceptor for subsequent progressive chain elongation by the sequential activities of WbdB, transferring successive mannosyl units into the 3 position, and of WbdA, transferring successive mannosyl units at the 2 position of the previous mannose. On the basis of this scheme, the presence of α-1,2 and α-1,3 linkages in the LPS O-side-chain of *Brucella *suggests the existence of at least two N-formyl-perosaminyltransferases, WbkA and WboA. The WbkA could interact with WboA to elongate the *Brucella *LPS O-side-chain by α-1,2 and α-1,3 links. The *wboA *gene that encodes a glycosyltransferase, an enzyme also essential for the biosynthesis of the O-side chain in *B. abortus *was characterized by McQuiston *et al. *[35]. The disruption of the *wboA *gene in smooth strains *B. abortus *2308, *B. melitensis *16 M and *B. suis *biovar 4 resulted in conversion to a rough phenotype and attenuated [28]. Vemulapalli *et al. *[36] discovered that the *wboA *gene is interrupted by an *IS711 *element in *B abortus *vaccine strain RB51. The complementation of RB51 with a functional *wboA *gene resulted in O-antigen production but did not result in reversion to the smooth phenotype and did not affect attenuation, suggesting that RB51 contains an additional genetic mutation(s) that probably affects either the export of O-antigen to the bacterial surface, the coupling of O-antigen to core lipopolysaccharide, or both [37]. Two rough mutant strains RA1 and VTRM1 derived from virulent *B. abortus *2308 or *B. melitensis *16 M, respectively have identical mutations *wboA *gene. RA1 strain was more sensitive to the bactericidal action of nonimmune human serum and more complement components were deposited on its surface than on strain VTRM1 [38]. Similar species-specific differences in both complement deposition and complement-mediated killing were also observed when strain RA1 was compared with another rough mutant of *B. melitensis*, WRR51. Strain WRR51 was derived from *B. melitensis *strain 16 M by replacement of the internal region of the *wboA *gene with an antibiotic resistance cassette instead of having a transposon insertion on this gene, as in the case of VTRM1 or RA1. There were no significant differences in either complement deposition or killing between VTRM1 and WRR51 [39]. Both strains were less susceptible than RA1 to the deposition of complement and complement-mediated killing. The LPS of strains RA1 and RB51 with the LPS of strain 2308 were compared and silver staining indicated that no O-side chain was associated with LPS of strains RA1 or RB51, and compositional analysis of smooth and rough *B. abortus *LPS revealed that 2-keto-3-deoxy-D-manno-2-octulosonic acid (KDO) was the predominant glycose in the rough LPS [35]. Vizcaíno *et al. *[40] studying DNA polymorphism in the *omp25/omp31 *family of *Brucella spp*., identified a 15.1 kb fragment absent in *Brucella ovis*. The region absent from *B. ovis *suggests that this DNA fragment is a genomic island acquired by the *Brucella *ancestor by horizontal transfer and later deleted from *B. ovis*. This deletion includes *wboA *and two other genes that might be involved in the LPS synthesis. Absence of these genes in *B. ovis *may explain, at least in part, the rough phenotype naturally displayed by this *Brucella *species. The complementation of rough *B. ovis *PA with plasmids bearing *wboA*, bearing *wboA *and the downstream gene potentially encoding a mannosyltransferase or bearing almost the entire 15.1 kb DNA fragment deleted in *B. ovis *strains did not confer a smooth phenotype, as shown by the lack of reactivity with a MAb specific for the *Brucella spp *S-LPS. This finding suggests that other genes required for the synthesis of S-LPS located at other chromosomal loci are affected in *B. ovis*. It seems clear that removal of the 15.1 kb genomic island from the smooth *Brucella *strains would reduce their virulence, since it was shown that the Tn*5*-disruption of *wboA *reduces survival of *B. abortus *in mice [35]. It would be interesting to determine how the complementation of *B. ovis *with the deleted genomic island would affect the virulence of this *Brucella *species.

### Phosphoglucomutase (pgm)

The gene encoding for phosphoglucomutase (*pgm*) is involved in O-antigen biosynthesis in *B. abortus *[41]. This gene is absolutely necessary for the biosynthesis of ADP-glucose, UDP-glucose, and UDP-galactose, the donors of glucose or galactose for the biosynthesis of molecules containing these sugars. The predicted protein is 74.7% identical to its homologue in *Agrobacterium tumefaciens *but is not part of the glycogen operon as it is in *Agrobacterium. B. abortus *LPS O-antigen is a homopolymer of perosamine, a derivative of mannose that is synthesized through GDP-mannose, thus, a *pgm *mutant of this species would not be impaired in the synthesis of GDP-perosamine, the sugar donor of O-antigen subunits. Insertional mutagenesis of *pgm *was carried out introducing a gentamicin-resistant gene within the *B. abortus pgm *gene and the electrophoretic profile of the LPS extracted from this mutant strain indicated lack of the O-antigen. This mutant was unable to survive in mice but replicates in HeLa cells, indicating that the complete LPS is not essential either for invasion or for intracellular multiplication. This behavior suggests that the LPS may play a role in extracellular survival in the animal, probably protecting the bacteria against complement-mediated lysis, but is not involved in intracellular survival. The fact that the mutant replicates at a lower rate is not necessarily a consequence of the rough phenotype, since the absence of *pgm *affects many other components of the cell wall, such as, for example, the synthesis of β (1,2) cyclic glucan [42]. Sequence analysis of the regions upstream and downstream of *Brucella pgm *revealed no significant homology to any gene in the database, which was surprising since in *A. tumefaciens *and *Rhizobium loti*, *pmg *is part of the glycogen operon.

### ABC type transporters (Wzm and Wzt)

Godfroid *et al*. [43] identified, sequenced and characterized a chromosomal locus of a 14-kb, *wbk *biosynthesis gene cluster, involved in the LPS O-side-chain biosynthesis of *B. melitensis *16 M. Analysis of the nucleotide sequence revealed the presence of seven open reading frames (ORFs), with six of them showing homology with genes involved in LPS O-side-chain biosynthesis from other organisms, and surrounded by four entire and one partial insertion sequences (IS). The seven ORFs were named according to the bacterial polysaccharide gene nomenclature proposed by Reeves *et al*. [44]. The GCG Gap program (Wisconsin package version 9.1, Genetic Computer Group, Madison, WI) was used to compare the deduced gene products and their similarity to various protein homologues. Seven genes of the *wbk *locus of *Brucella melitensis *16 M were *wbkA*, *gmd*, *per*, *wzm*, *wzt*, *wbkB*, and *wbkC*, coding, respectively, for proteins homologous to N-formyl-perosaminyltransferase, GDP-mannose 4,6 dehydratase, perosamine synthetase, ABC-type transporter (integral membrane protein), ABC-type transporter (ATPase domain), a hypothetical protein of unknown function, and a putative formyl transferase [45]. The *wzm *and *wzt *(putative the integral membrane component of ABC transporters) mutation resulted in a rough phenotype of *B. melitensis *16 M colonies as shown by crystal violet colony staining [43]. The *wzm*/*wzt *mutant also failed to react in ELISA on whole cells with MAbs directed against the S-LPS O-side-chain of *Brucella *species (anti-S-LPS MAbs), confirming the absence of the O-side-chain on the bacterial surface. Three complete and one incomplete insertion sequences in close association with the *wbk *gene cluster were found (IS*Bm1*, IS*Bm*, IS*Bm3*, IS*Bm*4). The presence of several ISs in intimate association with the *wbk *locus is intriguing. Since IS elements are suggested to play an important evolutionary role in mediating chromosomal rearrangements, it seems likely that they might have contributed to the structural evolution and probably horizontal acquisition of the O-antigen biosynthesis gene cluster in *Brucella spp*.

### Mannose (manA, B, C)

Monreal *et al. *[46] showed that several LPS genes flank the seven genes described by Godfroid *et al*.[43]. These genes include *manA*, *manB*, and *manC*, and their position strongly suggests that they act coordinately with *gmd *and *per *and independently of other mannose genes. Polymyxin B sensitive mutants were isolated by transposon mutagenesis of *B. abortus *2308 and screening for viability loss after a controlled exposure to an excess of this antibiotic [47]. Since the O-polysaccharide plays a role in protection against polymyxin B, these mutants were further screened for O-polysaccharide defects by agglutination with anti-S-LPS antibodies. Four mutants negative in this test were then chosen on the basis of their different polymyxin B sensitivities. Computer database analysis revealed that the mini-Tn*5 *was inserted in the *per *gene, *wbkA*, *manB *and open reading frame provisionally named wa**. This gene product was a membrane protein of the glycosyltransferase family involved in LPS biosynthesis, but it was different from other putative glycosyltransferases described before as involved in LPS synthesis in *Brucella*. Monreal *et al. *[46] did a search in the complete genome sequence of *B. melitensis *16 M and *B. suis *1330 and revealed a single homologous genes for *per *and *wbkA*, both located in the *wbk *region. The gene homologous to wa** was also in chromosome I, although in a different region. On the other hand, the *B. melitensis *and *B. suis manB *homologues were in chromosome II, along with a *manC *gene putatively coding for both mannose-6-P-isomerase and mannose-1-P-guanylyltransferase activities. Since phenotypic analysis revealed a severe core defect in the mutant, the gene was designated *manB*_*core*_. The genes wa** and *manB*_*core *_are involved in the biosynthesis of the *B. abortus *LPS core. In contrast to the parental strain, *per*, *wbkA*, wa**, and *manB*_*core *_mutants were resistant to the S-*Brucella*-specific phages and sensitive to the R-*Brucella*-specific phage R/C. Moreover, it was observed that the *manB*_*core *_mutant showed the lowest R/C phage sensitivity and the highest polymyxin B resistance and that, conversely, the wa** mutant had the highest R/C phage sensitivity and the lowest polymyxin B resistance.

Besides the antigen-O, the lipid A is the part of the LPS molecule responsible for its endotoxic activity. In *B. melitensis *16 M (Table 2) and *B. suis *(Table 3) were already identified some genes involved in lipid A biosynthesis, however mutants for those genes have not been described what makes harder to define the role of these gene products in LPS synthesis.

**Table 2:** Genes encoding lipid A biosynthesis in *Brucella melitensis*16 M.

Gene	Product	Location/Chromosome I/II
LpxA	Acyl-(acyl carrier protein) UDP-N-acetylglucosamine-O acyltransferase	856881 – 857729/I
LpxC	UDP-3-O-(3Hydroxymyristoyl) N-acetylglucosamine diacetylase	608267 – 609127/I
LpxD	UDP-3-O-(3Hydroxymyristoyl) glucosamine N-acetyltransferase	855363 – 856418/I
LpxB	Lipid-A-disaccharide synthetase	858609 – 859796/I
LpxK	Tetraacyldisaccharide-1-P4'-Kinase	1067291 – 1068316/II
KdsA	2-dehydro-3-deoxyphosphooctonate aldolase	876651 – 877448/I
KdsB	3-deoxy-manno octulosanate cytidylyl transferase	1959311 – 19601981/I
KdtA	3-deoxy-D-manno octulosonic-acid transferase	1068319 – 1069659/II
HtrB	Lauroyl/myristoyl acyltransferase	1159853 – 1160776/I

**Table 3:** Genes encoding lipid A biosynthesis in *Brucella suis*.

Gene	Product	Location/Chromosome I/II
LpxA	Acyl-(acyl carrier protein) UDP-N-acetylglucosamine-O acyltransferase	1130902 – 1131738/I
LpxC	UDP-3-O-Acyl-N-acetylglucosamine diacetylase	1379039 – 1379899/I
LpxD	UDP-3-O-(3Hydroxymyristoyl) glucosamine N-acetyltransferase	1132213 – 1133268/I
LpxB	Lipid-A-disaccharide synthetase	1128835 – 1130022/I
LpxK	Tetraacyldisaccharide-1-P4'-Kinase	202326 – 203351/II
KdsA	2-dehydro-3-deoxyphosphooctonate aldolase	1111145 – 1111978/I
KdsB	3-deoxy-manno octulosanate cytidylyl transferase	43162 – 43917/I
KdtA	3-deoxy-D-manno octulosonic-acid transferase	200983 – 202323/II
HtrB	Lauroyl acyltransferase	824862 – 825788/I

## Interaction between *Brucella *LPS and host innate immunity

In contrast to other intracellular pathogens, *Brucella *species do not produce exotoxins, antiphagocytic capsules or thick cell walls, resistant forms or fimbriae and do not show antigenic variation [48]. A key aspect of the virulence of *Brucella *is its ability to proliferate within professional and non-professional phagocytic host cells. Therefore, *Brucella *successfully bypasses the bactericidal effects of phagocytes, and their virulence and chronic infections are thought to be due to their ability to avoid the killing mechanisms of host cells [49]. Some studies with non-professional phagocytes have shown that *Brucella *invades host cells and is contained within early endosome-like vacuoles. These vacuoles rapidly fuse with early autophagosomes that acquire vacuolar H+-ATPase and lysosome-associated membrane proteins (LAMP) maturing into a late autophagosome. These autophagosomes inhibit fusion with lysosomes and finally become a replicating vacuole normally associated with the endoplasmic reticulum [50,51]. Porte *et al*. showed that the LPS O-side chain is involved in inhibition of the early fusion between *Brucella suis *containing phagosomes and lysosomes in murine macrophages at least during the first few hours after phagocytosis [52]. In contrast, the phagosomes containing rough mutants, which fail to express the O-antigen, rapidly fuse with lysosomes. The LPS O-chain might be a major factor that governs the early behavior of bacteria inside macrophages.

Recognition of the presence of LPS by cells such as monocytes and macrophages has evolved over centuries to provide the mammalian host with a rapid recognition of and reaction towards Gram-negative infection. This rapid, innate response against LPS typically involves the release of a range of pro-inflammatory mediators, such as TNF-α, IL-6, IL-12 and IL-1β, which in local sites of infection and in moderate levels benefit the host greatly by promoting inflammation and otherwise priming the immune system to eliminate the invading organisms. However, in conditions where the body is exposed to LPS excessively or systemically (as when LPS enters the blood stream), a systemic inflammatory reaction can occur, leading to multiple organ failure, shock and potentially death [53].

Recognition of bacterial LPS is mediated by CD14, however, CD14 lacks transmembrane and intracellular domains necessary for signal transduction and thus requires the involvement of molecules belonging to the TLR family. The recent discovery of TLR proteins, a family of mammalian pattern recognition receptors, has provided new insights into our understanding of the mechanisms by which *Brucella *can elicit cellular responses from innate immune cells. *B. abortus *induces interleukin (IL)-12 production from human monocytes and this effect was blocked by anti-CD14 antibody, suggesting that the *Brucella *binding and/or signaling to monocytes was mediated via LPS [54]. Additionally, *Brucella*'s ability to elicit IL-12 secretion enables it to drive Th0 cells to differentiate into Th1 effector and memory cells that are a central feature of the potential use of *B. abortus *as a vaccine carrier and adjuvant.

**Figure 2:**
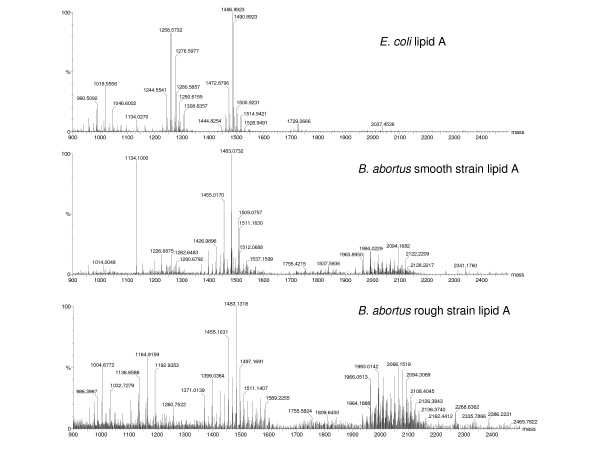
Mass spectrometry analysis of lipid A species from *Brucella abortus *smooth and rough strains and from *Escherichia coli*.

Our group has investigated the ability of *B. abortus *and its purified LPS and lipid A to trigger TLR2 and TLR4 and the impact of this activation in innate recognition and elimination of invading bacteria [55]. CHO reporter cell lines transfected with CD14 and TLRs showed that *B. abortus *triggers both TLR2 and TLR4. In contrast, lipopolysaccharide (LPS) and lipid A derived from *Brucella *rough (R) and smooth (S) strains activate CHO cells only through TLR4. Consistently, macrophages from C3H/HePas mice exposed to R and S strains and their LPS produced higher levels of tumor necrosis factor alpha (TNF-α) and interleukin-12 compared to C3H/HeJ, a TLR4 mutant mouse. The essential role of TLR4 for induction of proinflammatory cytokines was confirmed with diphosphoryl lipid A from *Rhodobacter sphaeroides*. Furthermore, to determine the contribution of TLR2 and TLR4 in bacterial clearance, numbers of *Brucella *were monitored in the spleen of C3H/HeJ, C3H/HePas, TLR2 knockout, and wild-type mice at 1, 3, and 6 weeks following *B. abortus *infection. Interestingly, murine brucellosis was markedly exacerbated at weeks 3 and 6 after infection in animals that lacked functional TLR4 (C3H/HeJ) compared to C3H/HePas that paralleled the reduced gamma interferon production by this mouse strain. By mass spectrometry analysis we found dramatic differences on the lipid A profiles of R and S *Brucella *strains when compared to *E. coli *lipid A (Figure 2). As shown in Figure 2, *Brucella *rough and smooth lipid A possess more heptaacylated species than *E. coli *lipid A that presents more hexaacylated residues. In conclusion, these results indicate that TLR4 plays a role in resistance to *B. abortus *infection. In contrast, Giambartolomei *et al *[56] claimed that lipoproteins Omp19 and Omp16 signaling through TLR2 rather than LPS are the key molecules involved in the proinflammatory response mediated by IL-6 and TNF-α observed during *Brucella *infection. This phenomenon seems to be related to the lipid moiety of the proteins. Therefore, TLR2 and TLR4 signaling during *Brucella *infection are mediated by two different ligands, lipoproteins and LPS, respectively. More recently, Weiss *et al*. [57] found that during infection of macrophages, *Brucella *avoids activation of TLR4 at six hours but activates TLR4, TLR2, and MyD88 (myeloid differentiation factor 88) at 24 hours postinfection. Interestingly, even though its activation is delayed, among the three MyD88 is the most important molecule for host defense against *Brucella *infection in vivo, since MyD88 knockout (KO) mice are more susceptible to infection when compared to TLR4, TLR2, TLR4/TLR2 KO mice. These findings open the possibility for the involvement of other TLRs that signal through MyD88 in host resistance against *Brucella *infection. As a matter of fact, Huang *et al. *[58] demonstrated that HKBA (heat-killed *Brucella abortus*) stimulates dendritic cells (DC) via TLR9 to secrete IL-12. Furthermore, this effect can be stimulated by DNA derived from HKBA and can be blocked by addition of suppressive oligodeoxynucleotides (ODNs). The consequence of TLR9 engagement is that Th1-like responses such as IFN-g production takes place in wild-type but not TLR9 KO mice.
